# Antioxidant Activities of *Streptomyces* sp. strain MUSC 14 from Mangrove Forest Soil in Malaysia

**DOI:** 10.1155/2020/6402607

**Published:** 2020-03-11

**Authors:** Hefa Mangzira Kemung, Loh Teng-Hern Tan, Kok-Gan Chan, Hooi-Leng Ser, Jodi Woan-Fei Law, Learn-Han Lee, Bey-Hing Goh

**Affiliations:** ^1^Biofunctional Molecule Exploratory Research Group (BMEX), School of Pharmacy, Monash University Malaysia, 47500 Bandar Sunway, Subang Jaya, Selangor Darul Ehsan, Malaysia; ^2^Novel Bacteria and Drug Discovery Research Group (NBDD), Microbiome and Bioresource Research Strength (MBRS), Jeffrey Cheah School of Medicine and Health Sciences, Monash University Malaysia, 47500 Bandar Sunway, Subang Jaya, Selangor Darul Ehsan, Malaysia; ^3^Institute of Biomedical and Pharmaceutical Sciences, Guangdong University of Technology, Guangzhou 510006, China; ^4^Division of Genetics and Molecular Biology, Institute of Biological Sciences, Faculty of Science, University of Malaya, 50603 Kuala Lumpur, Malaysia; ^5^International Genome Centre, Jiangsu University, Zhenjiang 212013, China; ^6^College of Pharmaceutical Sciences, Zhejiang University, Hangzhou 310058, China; ^7^Health and Well-Being Cluster, Global Asia in the 21st Century (GA21) Platform, Monash University Malaysia, Bandar Sunway 47500, Subang Jaya, Malaysia

## Abstract

The mangrove ecosystem of Malaysia remains yet to be fully explored for potential microbes that produce biologically active metabolites. In the present study, a mangrove-derived *Streptomyces* sp. strain MUSC 14 previously isolated from the state of Pahang, Malaysia Peninsula, was studied for its potential in producing antioxidant metabolites. The identity of *Streptomyces* sp. strain MUSC14 was consistent with the genotypic and phenotypic characteristics of the *Streptomyces* genus. The antioxidant potential of *Streptomyces* sp. strain MUSC 14 was determined through screening of its methanolic extract against sets of antioxidant assays. The results were indicative of *Streptomyces* sp. strain MUSC 14 displaying strong antioxidant activity against ABTS, DPPH free radicals and metal chelating activity of 62.71 ± 3.30%, 24.71 ± 2.22%, and 55.82 ± 2.35%, respectively. The result of ferric reducing activity measured in terms of dose was equivalent to 2.35–2.45 *μ*g of positive control ascorbic acid. Furthermore, there was a high correlation between the total phenolic content and the antioxidant activities with *r* = 0.979, *r* = 0.858, and *r* = 0.983 representing ABTS, DPPH, and metal chelation, respectively. Overall, the present study suggests that *Streptomyces* sp. strain MUSC 14 from mangrove forest soil has potential to produce antioxidant metabolites that can be further exploited for therapeutic application.

## 1. Introduction

Reactive oxygen species (ROS) are free radicals that tend to react with electron rich species at the cellular level in biological system causing cellular damage to their structure and function [1]. When produced at normal levels, the endogenous ROS is an important signalling molecule required by the immune system, to counter foreign substances including infectious bacteria [1]. During this process, an abnormal accumulation of free radicals may likely occur. As a result, the body produces antioxidants that can counter the effects of ROS. These antioxidants are enzymes such as catalase, superoxide dismutase, and glutathione peroxidase [1]. At higher levels of ROS, the production of endogenous antioxidants does not suffice to neutralize ROS [2]. A further supplement of antioxidants is necessary to alleviate high levels of ROS production in order to prevent deleterious effects on the cells, tissues, and organs which are associated with diseases such as cancer, diabetes, and cardiovascular and neurodegenerative diseases [2]. Other antioxidants are derived from food sources [3, 4], plants [5–8], and microbes [9–13].

To date, *Streptomyces* remains the most widely studied bacterial genus [14]. Currently there are about 852 validly published species and 38 subspecies recorded in the bacteria database [14]. According to Bérdy [15], there are an estimated 7600 biologically active compounds produced by *Streptomyces* alone. Furthermore, many drugs have been derived [16] from or inspired by natural products originating from *Streptomyces* genus [17]. The earliest discovery of *Streptomyces*-derived drugs dates back to the year 1940, when the antituberculosis drug streptomycin was isolated from *Streptomyces griseus* [18]. Since then, numerous drugs have been discovered from *Streptomyces* [16]. However, the pace of drug discovery from *Streptomyces* seems to have dwindled in recent years [19] owing mainly to rediscovery of compounds. For example, streptomycin, tetracycline, and actinomycin D produced by *Streptomyces* have a frequency of occurrence of 100^−1^. In other words, there is a possibility of recovering one of these compounds when conducting random screening of 100 strains from given soil samples [20]. In fact, many of these previously discovered drugs were isolated from *Streptomyces* from soil samples. According to the retrospective analysis by Pye et al. [21], there is a notable decrease in percentage in discovery rate of novel compounds, despite observing an increase in natural products and natural product research interest worldwide [21]. Equally important was the finding that suggests novel sources of natural product as reflective of novel chemical diversity [21, 22]. As such, researchers have begun to shift their focus to understudied ecological niches such as mangrove ecosystem [23], consequently in the hope of discovering new sources and hence unique chemistry and biological activities.

The mangrove ecosystem is a thriving habitat of trees and shrubs situated in intertidal zones and estuaries mainly along the tropical and subtropical coastlines [24]. It is postulated that organisms residing therein have developed unique metabolic pathways that would allow them to tolerate harsh environmental condition such as higher-than-normal levels of salinity and fluctuations in tidal gradients, temperature, and pH. The microbial community is no exception. The fact that microbial communities in mangrove ecosystem, particularly of soil, remain yet to be studied [25] provides the impetus for drugs discovery research focused on *Streptomyces* genus. A larger proportion of the microbial community are bacteria and fungi that constitute an estimated 91% of microbial biomass [26]. Interestingly, the mangrove ecosystem in South-East Asia holds the largest coverage of mangrove forests [27]. Because Malaysia is ideally located within the South-East Asian region, it offers an opportunity to explore its microbial community as it remains yet to be fully explored. Recent review by Ser et al. [23] detailed the potential of *Streptomyces* from mangrove soil in Malaysia to produce biologically active metabolites with anticancer and antioxidant activities with addition to studies carried out by Kemung et al. [10], Tan et al. [28–30] and Law et al. [31]. Meanwhile study by Ser et al. [32] demonstrated *Streptomyces* as potential producers of antibacterial metabolites. Altogether, these studies support the notion that *Streptomyces* from harsh environment have the potential to produce biologically active metabolites that could be of therapeutic benefit to treat various diseases.

Given that previously isolated *Streptomyces* from mangrove soil have shown promising biological activities including antioxidant activity, this study attempts to investigate the potential of *Streptomyces *sp. strain MUSC 14 isolated from mangrove soil in Pahang, Peninsular Malaysia. We employed 4 antioxidant tests to determine the antioxidant potentials of the methanolic extract prepared from *Streptomyces* sp. strain MUSC 14. Moreover, the total phenolic content of the methanolic extract was investigated in an attempt to correlate the antioxidant activities with the presence of the phenolic content of the extract.

## 2. Materials and Methods

### 2.1. Test Bacteria and Maintenance

The bacteria strain labelled MUSC 14 (Monash University Sunway Campus strain number 14) was previously isolated from soil collected in mangrove forest (MUSC-TLS4 3°48′21.3″ N 103°20′3.3″E) of Tanjung Lumpur, in the State of Pahang, Peninsular Malaysia, on December 2012 [33]. Pure cultures of strain MUSC 14 were selected and maintained on ISP2 agar slant at 28°C and glycerol stocks (30% v/v) at −80°C for long term preservation [29, 34].

### 2.2. Genomic and Phylogenetic Analysis of Strain MUSC 14

The genomic DNA (gDNA) was extracted following the protocol as described by Hong et al. [35]. The amplification of 16S rRNA gene was performed according to Lee et al. [36]. Briefly, PCR reactions were run in a final volume of 50 *μ*L on Kyratex PCR Supercycler (Kyratex, Australia) according to SolGentR protocol with cycling conditions: (i) 95°C for 5 mins; (ii) 35 cycles of 94°C for 50 s, 55°C for 1 min, and 72°C for 1 min 30 s; (iii) 72°C for 8 mins. The 16S rRNA gene sequence was aligned with related type *Streptomyces* strains accessible on GenBank/EMBL/DDBJ databases using CLUSTAL-X software [37]. A phylogenetic tree was constructed by neighbour-joining method [38] using MEGA version 6.0 software [39]. Kimura's two-parameter model for the neighbour-joining algorithms was used to establish evolutionary distances [40]. The EzBiocloud (previously known as ez-Taxon) database was used to confirm sequence similarities [41]. Stability of generated phylogenetic trees was tested using bootstrap based on 1000 resampling method [42].

### 2.3. Phenotypic Characterization of Strain MUSC 14

Strain MUSC 14 was incubated for a period of 7–14 days at 28°C and its growth and cultural characteristics on different culture media were determined. In brief, the growth on culture media and production of soluble pigment were observed on agars—International *Streptomyces* Project (ISP) 2, ISP3, ISP4, ISP5, ISP6, ISP7 [43], *Streptomyces* agar (SA) [44], nutrient agar (NA) [45], actinomycete isolation agar (AIA) [46], and starch casein agar (SCA) [47]. Colour of colony was described using standard ISCC-BS colour charts [48]. The strain MUSC 14 was also subjected to different growth temperature ranging from 4°C to as high as 50°C. Tolerance to NaCl was demonstrated using concentrations 0–10% with an interval of 2%. The pH conducive for growth in Tryptone Soya Broth (TSB) culture media [49] was determined using pH scale from 2 to 10 [11, 28]. Microscopic cell morphology of a 7–14-day-old culture grown on ISP 2 solid media was observed under JEOL-JSM 6400 scanning electron microscope. Catalase test was done by adding a drop of 3% (v/v) hydrogen peroxide to the culture. Production of bubbles indicated positive for catalase activity [50]. To test for haemolytic activity, a 5-day-old culture was grown on blood agar media with ingredients 5% (w/v) peptone, 3% (w/v) yeast extract, 5% (w/v) NaCl, and 5% (v/v) human blood [51]. Clear zone around the 5-day-culture indicated haemolysis and surfactant property of the culture. Presence of exoenzymes chitinase, xylanase, amylase, protease, lipase, and cellulose was determined by growing culture on ISP 2 media and following protocol as described by Meena et al. [52].

### 2.4. Fermentation and Extract Preparation of Strain MUSC 14

The strain MUSC 14 was revived in 10 mL TSB as seed culture for fermentation and maintained on ISP2 agar at room temperature of 28°C. A pure culture colony on ISP2 agar was selected and transferred to 10 mL sterile TSB and incubated at 28°C, 200 rpm for 10 days. The tubes were shaken at tilted position to allow for optimal aeration. 1 mL of seed culture was transferred to 200 mL Han's Fermentation Media 1 (HFM1) in a 500 mL conical flask and grown for 10 days at 28°C with aeration rate of 200 rpm. Fermented broth was centrifuged at 4000 rpm at 4°C for 5 mins and filtered using Whatman filter paper [36]. The supernatant was subjected to freeze drying process. Twenty millilitres of supernatant was transferred into 50 mL tubes and freeze-dried for 3 days at −45°C. Extraction of freeze-dried samples was performed using absolute methanol as the solvent of extraction and carried out three times. Filtration of extract was performed using Whatman filter paper. Filtrate collected was concentrated using a rotary vacuum evaporator and kept at −20°C until further analysis [29].

### 2.5. Antioxidant Potential of Extract MUSC 14

#### 2.5.1. ABTS Radical Scavenging Activity

The 2,2′-azino-bis(3-ethylbenzothiazoline-6-sulfonic acid) (ABTS) radical scavenging assay was performed as described previously by Tan et al. [28]. In brief, stable ABTS radical cation (ABTS•+) were first generated by mixing 7 mM of ABTS stock solution with potassium persulfate at 2.45 mM. The premade ABTS free radical solution was then added to 96-well plates containing concentrations of MUSC 14 extract ranging from 0.125 mg/mL, 0.25 mg/mL, 0.5 mg/mL, 1 mg/mL, 2 mg/mL, and 4 mg/mL. The plates were left at room temperature in the dark room for 20 minutes to allow the extract to react with the ABTS free radical cation. Gallic acid was used as the positive control. The absorbance reading was taken afterwards at 734 nm. A reduced absorbance reading indicated change in the amount of free radical present in the reaction mixture. The ABTS scavenging activity was expressed in percentage using the following equation:

(1) $$\begin{array}{c} \%\text{ ABTS scavenging activity}=\frac{\text{Absorbance of control}–\text{Absorbance of sample}}{\text{Absorbance of control}}\times 100\text{\%.} \end{array}$$

#### 2.5.2. DPPH Radical Scavenging Activity

The 2,2-diphenyl-1-picrylhydrazyl (DPPH) radical scavenging activity was carried out according to Tan et al. [28]. In brief, different concentrations of methanolic extract of MUSC 14 ranging from 0.125 mg/mL, 0.25 mg/mL, 0.5 mg/mL, 1 mg/mL, 2 mg/mL, and 4 mg/mL were mixed with premade DPPH (0.016% w/v) in absolute ethanol (95% v/v). The reaction mixture was left in the dark for 20 minutes at room temperature and afterwards the absorbance reading was taken at 515 nm using a microplate reader. Gallic acid was used as the positive control. The DPPH scavenging activity was expressed in percentage using the following equation:

(2) $$\begin{array}{c} \%\text{ DPPH radical scavenging activity}=\frac{\text{Absorbance of control}–\text{Absorbance of sample}}{\text{Absorbance of control}}\times 100\%. \end{array}$$

#### 2.5.3. Metal Chelating Activity

Metal chelating activity of the methanolic extract of MUSC 14 was determined following the methods described by Adjimani and Asare [53]. The extract of MUSC 14 was prepared in different concentrations by 2-fold dilution of 0.125 mg/mL, 0.25 mg/mL, 0.5 mg/mL, 1 mg/mL, 2 mg/mL, and 4 mg/mL and added to 96-well plates. Afterwards, ferrous sulfate (FeSO_4_) at 2 mM was added to each well followed by ferrozine at 5 mM. The reaction mixture was left to react at room temperature for 10 minutes. The absorbance was afterwards read at 562 nm. The metal chelating activity measures the ability of extract to interfere with the formation of ferrozine-ferrous ion complex. Ethylenediaminetetraacetic acid (EDTA) was used as the positive control. The metal chelating activity was expressed in percentage using the following equation:

(3) $$\begin{array}{c} \%\text{ Metal chelating activity}=\frac{\text{Absorbance of control}–\text{Absorbance of sample}}{\text{Absorbance of control}}\times 100\%. \end{array}$$

#### 2.5.4. Ferric Reducing Activity

The ferric reducing activity was performed as described by Adjimani and Asare [53] with some modifications. A volume of 25 *μ*L of varying concentrations for methanolic extract of MUSC 14 was prepared (2.5 mg/mL, 5 mg/mL, 10 mg/mL, 20 mg/mL, 40 mg/mL, and 80 mg/mL) and added to respective 1.5 mL tubes. Next, 25 *μ*L phosphate buffer (0.2 M) and 25 *μ*L (1%) of K_3_Fe(CN)_6_ were mixed with the extract. The reaction mixture was heated to 50°C and left to react for 20 minutes. This was then allowed to cool to room temperature before adding 25 *μ*L of TCA (10%) to stop the reaction. An aliquot of 80 *μ*L of the reaction mixture was added to 96-well plates followed by 20 *μ*L of FeCl_3_. The absorbance reading was taken at 700 nm. The ascorbic acid dose equivalents for methanolic extract were determined using equations (4) and (5). A standard curve derived from calibration of ascorbic acid (*R*^2^ = 0.97) with formula depicted in equation (4) was used to calculate the ascorbic acid equivalents in terms of concentration (mg/mL). This was followed by using the formula of concentration which equals the mass divided by the volume in order to determine the mass (dose) equivalent of ascorbic acid as shown in as shown in equation (5).

(4) $$\begin{array}{c} \text{Ascorbic acid equivalents }\left(\text{mg}|/|\text{mL}\right)=\frac{\text{Absorbance of sample}-0.4252}{4.9433}, \end{array}$$

(5) $$\begin{array}{c} \text{Ascorbic acid dose equivalents}\left(\text{mg}\right)=\frac{\text{Equation }\left(4\right)}{1000\;µ\text{L}}\times 25\;µ\text{L}. \end{array}$$

### 2.6. Total Phenolic Content Determination with Folin–Ciocalteu's Reagent Method

The total phenolic content (TPC) of the methanolic extract of MUSC 14 was determined following the method of Tan et al. [28]. The methanolic extract of MUSC 14 was prepared in a series of concentration ranging from 0.125 mg/mL, 0.25 mg/mL, 0.5 mg/mL, 1 mg/mL, 2 mg/mL, and 4 mg/mL at 10 *μ*L and subsequently added to 96 wells followed by 50 *μ*L of diluted Folin-Ciocalteu's Reagent (1 : 10). The reaction mixture was incubated for 5 minutes in the dark at room temperature. A volume of 40 *μ*L of sodium carbonate (NaCO_3_) at 7.5% was added afterwards and the reaction mixture further allowed to react for another 30 minutes at room temperature. The absorbance reading was taken at 750 nm using a microplate reader and the results obtained were expressed in Gallic acid equivalents.

### 2.7. Gas Chromatography-Mass Spectroscopy (GC-MS) Analysis of Methanolic Extract

The profiling of chemical constituents in the methanolic extract of strain MUSC 14 was carried out based on method described by Tan et al. [54]. Agilent Technologies 6980N was coupled with a 5979 Mass Selective Detector. The HP-5MS (5% phenyl methyl siloxane) capillary column of dimensions 30.0 m × 250 *μ*m × 0.25 *μ*m was used as helium gas carrier at 1 mL/min. Initial temperature of column was set at 40°C with a gradual increase of 3°C every minute until it achieved the maximum of 250°C and then stationed for an intermediate 5 minutes. MS was set to operate at 70 eV. The detected constituents were identified by comparing their mass spectral data with standard compounds from NIST 05 spectral Library.

### 2.8. Statistical Analysis

The tests were run in triplicate. The results were expressed in means ± standard deviation (SD). Statistical Package for the Social Sciences software (SPSS) was used to perform the statistical analysis for the antioxidant assays. One-way analysis of variance (ANOVA) together with Tukey's post hoc was used to determine the statistical significance with a *p* value <0.05. Pearson's correlation on SPSS software was used to determine the relationship between the total phenolic content in the methanolic extract and the respective antioxidant activity.

## 3. Results and Discussion

### 3.1. Genomic and Phylogenetic Analysis of Strain MUSC 14

**Figure 1:**
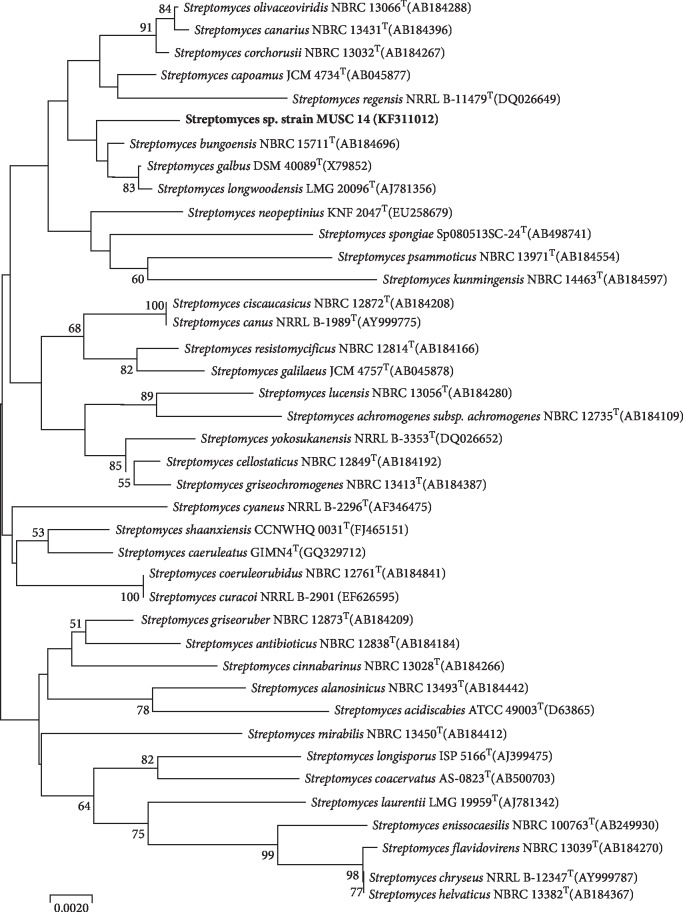
Neighbour-joining phylogenetic tree based on 1391 nucleotides of 16S rRNA gene sequence (KF311012) showing the relationship between strain MUSC 14 and representatives of related taxa. Numbers and nodes indicate percentages (>50%) of 1000 bootstrap resampling. Bar, 0.002 substitutions per site.

The phylogenetic analysis on the basis of the 16S rRNA led to the identification of strain MUSC 14 (GenBank/EMBL/DDBJ accession number KF311012) as belonging to the genus Streptomyces (Figure 1) and hence denoted *Streptomyces* sp. strain MUSC 14. The phylogenetic tree constructed for strain MUSC 14 on the basis of its 16S rRNA sequence revealed highest gene sequence similarity with *Streptomyces bungoensis* NBRC 15711^T^ and *Streptomyces galbus* 40089 DSM^T^ (99.35%) followed by *Streptomyces longwoodensis* LMG 20096^T^ (99.28%).

### 3.2. Phenotypic Characterization of Strain MUSC 14

**Figure 2:**
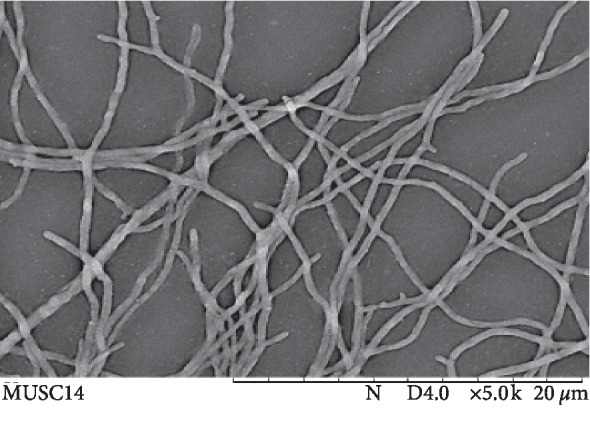
Scanning electron microscope showing the morphology of strain MUSC 14. This appears to be filamentous with extensive branching, which is a characteristic observed commonly in *Streptomyces*.

Phenotypic characterization provided additional information about the strain MUSC 14. On the basis of culture media used, it can be suggested that strain MUSC 14 preferred to grow on media containing yeast extract, malt extract, glycerol, asparagine, peptone, iron, tyrosine, and starch casein rather than peptone, sodium caseinate, and oatmeal. No growth on ISP4 media may suggest that MUSC strain requires additional nutrients such as sources of nitrogen apart from inorganic salts and soluble starch for growth (Table 1). The cell morphology of strain MUSC 14 was well developed and not fragmented after 7–14 days when grown on ISP 2 media and is consistent with strain type assigned to the *Streptomyces* genus (Figure 2) [55, 56]. Colony colour of both the aerial and substrate mycelia was present on all media tested except ISP4 (no growth observed). Colour of aerial mycelia is one of the important characteristics to categorize *Streptomyces* [57]. Strain MUSC14 exhibited different colony colour when grown on different media tested. Soluble pigments were absent on all media tested (Table 1).

Mangrove forests are mainly found in tropical and subtropical regions in the world and concentrated mostly in 15 countries including Malaysia [58]. The result of the physical tolerance levels suggested that strain MUSC 14 has adapted well to the mangrove ecosystem (Table 2). The strain MUSC 14 was able to grow within the temperature range of 26–50°C (optimal 26–37°C). Furthermore, it was able to withstand salinity levels up to 6% (w/v). The result indicated that strain MUSC 14 can tolerate up to a certain level of salinity which is necessary because of its exposure to the salty marine waters. Seawater is one of the factors that influence the pH of mangrove soil. Interestingly, the pH in mangrove soil varies among a number of mangrove forests studied thus far. For example, in Sibuti wildlife sanctuary situated in the state of Sarawak, Malaysia, the pH of mangrove forest soil was acidic (3.34) [59, 60], whilst the pH of mangrove soil in Pahang, Malaysia, was 6.1–6.4 at the time of measurement [33]. In Sundarbans mangrove forest, the soil was determined to be alkaline (7.2–8.4) [61]. In the present study, it was shown that strain MUSC 14 preferred to grow at pH 6-7 which shows that it adapted to the pH of mangrove forest in Pahang, Malaysia [33]. The results from biochemical test were positive for catalase, amylase, and cellulase (Table 2). *Streptomyces* are known as soil-dwelling saprophytic bacteria [56] that feed on dead and decaying matters. The extracellular enzymes that were shown to be produced by strain MUSC 14 suggest it was capable to degrade variety of materials and obtain its nutrients for growth and reproduction.

### 3.3. Antioxidant Activities of Extract MUSC 14

Several diseases are implicated with high levels of ROS in the body, including diabetes [62], cardiovascular disease, inflammation and arthritis [63], cancer [64], and neurodegeneration [65]. Furthermore, there seems to be growing interest in the use of naturally derived antioxidants rather than synthetic antioxidants by food industries [66].

In the fermentation of strain MUSC 14, the highly nutritious culture medium TSB contained pancreatic digest of casein and papaic digest of soybean meal as sources of nitrogen with dextrose as the source of carbohydrate for growth. To facilitate the production of secondary metabolites from strain MUSC 14, an aliquot of the seed culture was transferred to Han's Fermentation media 1 (HFM1) in 200 mL [28, 54], which is an optimized media that has been previously used to ferment *Streptomyces* from mangrove soil in Malaysia. HFM1 constitute the peptone, yeast extract, soluble starch, and calcium carbonate as sources of nitrogen, carbohydrates, and ions for growth.

The methanol is widely used as an organic solvent to carry out extraction of phenolic compounds and to determine their antioxidant activities. This is due to its amphiphilic nature and its better solvation power compared to ethanol at absolute concentration allowing methanol to attract greater quantities of nonpolar and polar compounds. For example, a study by Santas et al. [67] showed that white onion and calcot extracts obtained from pure methanol solvent gave the highest phenolic content and antioxidant activity when compared to absolute ethanol and acetone. This was also true in another study by Truong et al. [68] who showed that methanol was the best solvent of extraction used in their study with highest phenolic yield and strongest antioxidant activity.

Given that there are multiple known pathways responsible for inducing ROS as well as the nature of the methanolic crude extracts comprising a mixture of many compounds, this provides the basis to utilize a complementary sets of antioxidant assays [69, 70]. Thus, this study explored several antioxidant assays in order to assess the antioxidant capacity of the strain MUSC 14 extract (Table 3). Antioxidants can either prevent formation of ROS or scavenge ROS species. The former represents enzymes while the latter are nonenzymatic in nature. The antioxidant assays investigate the potential of nonenzymatic antioxidants to scavenge ROS species. Those that are rich in hydrogen atom, such as polyphenols, donate their hydrogen atom to ROS species in a process called hydrogen atom transfer (HAT) [69, 70] in an attempt to neutralize free radicals. Both ABTS and DPPH [71, 72] are commonly employed in antioxidant assays to determine the *in vitro* antioxidant capacity of natural products. Moreover, they are simple to perform and are highly sensitive methods and can provide a preliminary insight into the antioxidant capacity of natural products. Again, further tests are required to support results obtained from either DPPH or ABTS. In biological system, free radicals are harmful to biological cells and tissues as they can attack, for example, the lipids, proteins, and the delicate DNA structures leading to damaged cells, tissues, and organs which are prerequisite to the aforementioned diseases. Even though ABTS and DPPH assays do not prove relevant to biological systems [69] to a certain degree, they offer insight to the potential of natural products as antioxidants. The results of ABTS showed significant radical scavenging activity of extract MUSC 14 (*p* > 0.05) of 62.71 ± 3.30% at 4 mg/mL and 28.36 ± 4.72% at concentration of 2 mg/mL. Moreover, the DPPH radical scavenging activity (*p* > 0.05) of extract MUSC 14 was determined to be 24.71 ± 2.22% at 4 mg/mL.

There is a tendency for higher levels of iron circulating in the body to generate ROS species through the Fenton reaction [73–75]. In this reaction, the ferrous ion reduces hydrogen peroxide to ROS, which may consequently react with molecules in the body such as lipids [76, 77]. They have been implicated in increased levels of ROS and subsequent cellular and organ damage [78–80]. Studies have demonstrated that antioxidants from plants [81, 82] and microbes [28, 83] were effective in chelating with iron and thereby preventing formation of ROS. The result of this study showed that extract MUSC 14 had strong metal chelating activity at 55.82 ± 2.35% and 37.70 ± 2.03% at 4 mg/mL and 2 mg/mL, respectively. Overall, ABTS showed the strongest activity subsequently followed by metal chelating and DPPH activities. Previous studies reported similar trend of *Streptomyces* extracts having higher ABTS activities compared to the metal chelating and DPPH activities. For example, methanolic extract of *Streptomyces* sp. MUM212 from mangrove soil in Malaysia exhibited antioxidant activities of 61.52 ± 3.13%, 41.98 ± 0.73%, and 22.03 ± 3.01% against ABTS, chelating metal ions, and scavenging of DPPH radicals, respectively [28]. In another study, the methanolic extract of the mangrove-derived *Streptomyces* sp. MUSC 11 demonstrated the strongest antioxidant activity against ABTS radicals with 31.42 ± 1.00% compared to metal chelating and DPPH activities which were found to be of slightly lesser value with 21.61 ± 1.71% and 7.27 ± 4.73%, respectively [11]. The antioxidant activity of *Streptomyces* sp. MUM265 in terms of the results of ABTS, metal chelation, and DPPH radicals was 88.50 ± 0.37%, 46.02 ± 0.86%, and 42.33 ± 3.98%, respectively [54]. Given the varied results between ABTS, DPPH radicals, and metal chelation activities of methanolic extract of *Streptomyces* sp. MUSC 14, this suggests the importance of employing multiple antioxidant tests when determining the antioxidant potential of *Streptomyces* extracts.

### 3.4. Ferric Reducing Activity

**Figure 3:**
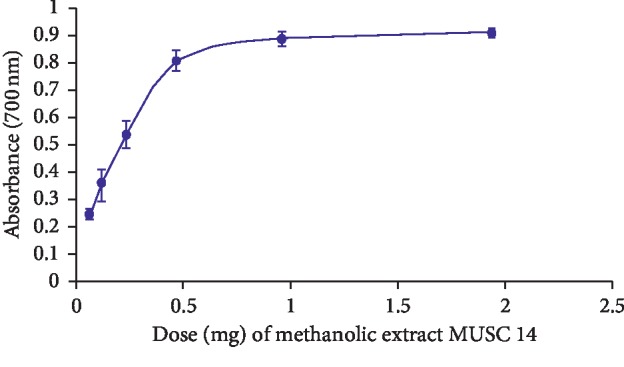
Ferric reducing activity of methanolic extract of strain MUSC 14. The error bars represent the standard deviation for calculation of the 6 doses (0.0625 mg, 0.125 mg, 0.25 mg, 0.5 mg, 1 mg, and 2 mg), used in the experiment. The experiment was run in triplicate (*n* = 3).

The ferric reducing assay determines the antioxidant activity of natural products by assessing the ability of a given natural product to reduce the ferric (Fe^3+^) ion to its ferrous (Fe^2+^) form. Unlike ABTS and DPPH assays that accept an electron transfer from an antioxidant supplier, the ferric reducing assay assesses the ability of natural product to undergo electron transfer reaction with the ferric ion [84]. In this study, extract MUSC 14 exhibited ferric reducing power at absorbance ranging from 0.89 ± 0.08 to 0.91 ± 0.02 which is in the dose range of 1 to 2 mg (Figure 3). Using equation (5), the given dose range of 1-2 mg was found to be equivalent to 2.35–2.45 *μ*g of ascorbic acid.

### 3.5. Total Phenolic Content

Over the years, interest in drug discovery studies of microbes has increased, leading researchers to investigate the antioxidant activities of microbial metabolites [85–87]. Among these metabolites, phenolic compounds, in particular, have been correlated well with antioxidant activities in several published reports [28, 54]. Hence, the strong antioxidant activity of MUSC 14 extract prompted further investigation of its phenolic content. The results for the total phenolic test show a strong correlation (*p* < 0.05) between the phenolic content and antioxidant activities of extract MUSC 14, suggesting that the antioxidant activity (ABTS, DPPH, and metal chelation) may most likely be due to the phenolic content present in the methanolic extract (Table 4).

### 3.6. GC-MS Analysis of MUSC 14 Extract

**Figure 4:**
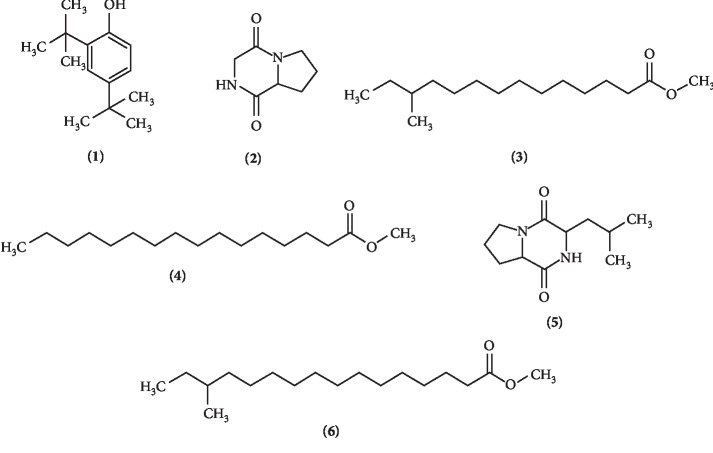
Chemical structures of the compounds identified in strain MUSC14 extract.

The GC-MS spectroscopy greatly assisted in determining the identity of chemical constituents present in the extract MUSC 14 (Table 5 and Figure 4). These individual compounds are as follows: phenol, 2,4-bis(1,1-dimethylethyl)- **(1)**, pyrrolo [1,2-a]pyrazine-1,4-dione, hexahydro **(2)**, tetradecanoic acid, 12-methyl-, methyl ester **(3)**, hexadecanoic acid, methyl ester **(4)**, pyrrolo [1,2-a]pyrazine-1,4-dione, hexahydro-3-(2-methylpropyl)- **(5)**, hexadecanoic acid, 14-methyl-, methyl ester **(6)**.

Among the phenolic compounds normally detected in fermentation broth of *Streptomyces,* phenol, 2,4-bis(1,1-dimethylethyl)- **(1)** is often associated with antioxidant activities [28, 29, 88–90]. Given that the results of total phenolic content showed a strong correlation with overall antioxidant activities, the findings of phenol, 2, 4-bis(1,1-dimethylethyl)- **(1)** in the extract of *Streptomyces* MUSC 14 strongly suggest playing a role in the antioxidant activities. Apart from its antioxidant property, compound **(1)** also displayed antifungal, antibacterial, and anticancer activity in *Vibrio alginolyticus* G16 and *Paracoccus pantotrophus* FMR19 [91, 92], respectively.

Pyrrolopyrazines are aromatic heterocyclic compounds consisting of an aromatic pyrazine fused with a pyrrole ring. Based on previous studies, this class of compounds is known to be produced by *Streptomyces* species. The presence of pyrrolo [1,2-a]pyrazine-1,4-dione, hexahydro **(2)** and pyrrolo [1,2-a]pyrazine-1,4-dione, hexahydro-3-(2-methylpropyl) **(5)** is no exception. Both of these compounds have appeared in extract of *Streptomyces,* previously studied [9, 10, 31, 54]. As such, it is possible to assume that pyrrolopyrazines may have contributed to the overall antioxidant activity observed in extract of strain MUSC 14.

A number of fatty acid esters were noted in the *Streptomyces* MUSC 14 extract. These are esters of fatty acids formed by reacting fatty acids with alcohols. The tetradecanoic acid, 12-methyl-, methyl ester **(3)** was found in the extract of *Streptomyces malaysiense* [90]. *Streptomyces cavouresis* KU-V39 produced hexadecanoic acid, methyl ester demonstrating antioxidant and anticancer activity **(4)** [93]. Meanwhile, hexadecanoic acid, 14-methyl-, methyl ester **(6)** was found in a *Streptomyces* extract with fumigant activity [94].

## 4. Conclusions

The study underscores the potential of *Streptomyces* sp. strain MUSC 14 as an exceptional producer of broad-spectrum antioxidant metabolite with ABTS, DPPH radical scavenging, metal chelating, and ferric reducing activity. Furthermore, there was strong correlation between the phenolic content and the antioxidant activities which suggests that the antioxidant activities of extract MUSC 14 were due to its phenolic content. In short, *Streptomyces* sp. strain MUSC 14 from mangrove soil in Pahang, Peninsular Malaysia, showed promising antioxidant activities that can be further explored for therapeutic application.

## Figures and Tables

**Table 1:** Cultural characteristics of strain MUSC 14.

Media	Growth	Colony colour		Soluble pigments
		Aerial mycelia	Substrate mycelia	
ISP 2	Well	Moderate yellow	Dark yellow	—
ISP 3	Poor	Yellowish grey	Yellowish grey	—
ISP 4	—	—	—	—
ISP 5	Well	Yellowish white	Yellowish white	—
ISP 6	Well	Greenish yellow	Dark olive brown	—
ISP 7	Well	Yellowish white	Yellowish white	—
AIA	Moderate	Yellowish white	Yellowish white	—
SCA	Well	Yellowish white	Yellowish white	—
SA	Well	Yellowish grey	Vivid yellow	—
NA	Moderate	Yellowish white	Light yellow	—

Key: (—): no growth and production of soluble pigment.

**Table 2:** Biochemical and physiological characteristics of strain MUSC 14.

Characteristics	Strain MUSC 14
Biochemical characteristics	
Catalase	+
Haemolytic activity	−

Enzymatic test	
Chitinase activity (2.5% chitin)	−
Xylanase activity (0.5% xylan)	−
Amylolytic activity (0.2% starch)	+
Protease activity (2% casein)	−
Lipase activity (1% tributyrin)	−
Cellulase activity (0.5% CMC)	+

Physiological characteristics	
Temperature (°C) tolerance	
Growth	26–50
Optimum	26–37

NaCl (%) tolerance	
Growth	0–6
Optimum	0–2

pH tolerance	
Growth	6-7
Optimum	6-7

Key: (+): activity; (−): no activity.

**Table 3:** The antioxidant activities of extract MUSC 14 at different antioxidant assay.

Concentration (mg/mL)	Antioxidant activities (%)		
	DPPH radical scavenging activity (%)	ABTS radical scavenging activity (%)	Metal chelating activity (%)
0.125	2.74 ± 1.40^*∗*^	5.40 ± 0.36^*∗*^	22.86 ± 0.60^*∗*^
0.25	2.38 ± 1.80^*∗*^	5.76 ± 0.89^*∗*^	20.94 ± 2.54^*∗*^
0.5	7.16 ± 1.58^*∗*^	9.23 ± 0.66^*∗*^	23.57 ± 1.39^*∗*^
1	11.88 ± 2.92^*∗*^	15.29 ± 1.27^*∗*^	30.83 ± 1.72^*∗*^
2	28.14 ± 2.80^*∗*^	28.36 ± 4.72^*∗*^	37.70 ± 2.03^*∗*^
4	24.71 ± 2.22^*∗*^	62.71 ± 3.30^*∗*^	55.82 ± 2.35^*∗*^
Gallic acid^a^	53.99 ± 4.06^*∗*^	—	—
Gallic acid^b^	—	42.50 ± 0.60^*∗*^	—
EDTA^c^	—	—	68.49 ± 7.68^*∗*^

^*∗*^Statistically significant at *p* < 0.05. ^a^Activity of gallic acid at 10 *μ*g/mL. ^b^Activity of gallic acid at 12.5 *μ*g/mL. ^c^Activity of EDTA at 0.125 mg/mL.

**Table 4:** Total phenolic content of methanolic extract MUSC 14.

Antioxidant activities	Phenolic content
ABTS radical scavenging activity	*r* = 0.979^*∗*^
DPPH radical scavenging activity	*r* = 0.858^*∗*^
Metal chelating activity	*r* = 0.983^*∗*^

^*∗*^Correlation is significant at the 0.05 level.

**Table 5:** Compounds identified in the methanolic extract MUSC 14 through GC-MS.

No.	Constituents	Retention time (min)	Molecular formula	Molecular weight	Similarity (%)
1	Phenol,2,4-bis(1,1-dimethylethyl)-	44.145	C_14_H_22_O	206	85.3
2	Pyrrolo[1,2-a]pyrazine-1,4-dione, hexahydro	53.563	C_7_H_10_N_2_O_2_	54	95.3
3	Tetradecanoic acid, 12-methyl-, methyl ester	54.495	C_16_H_32_O_2_	256	91.5
4	Hexadecanoic acid, methyl ester	57.594	C_17_H_34_O_2_	270	96.3
5	Pyrrolo[1,2-a]pyrazine-1,4-dione, hexahydro-3-(2-methylpropyl)-	59.404	C_11_H_18_N_2_O_2_	210	91.6
6	Hexadecanoic acid, 14-methyl-, methyl ester	60.813	C_18_H_36_O_2_	284	84.5

## Data Availability

The 16SrRNA gene sequence of strain MUSC 14 has been deposited in the GenBank repository with accession number KF311012.
